# Dissection of Mechanisms of a Chinese Medicinal Formula: Danhong Injection Therapy for Myocardial Ischemia/Reperfusion Injury *In Vivo* and *In Vitro*

**DOI:** 10.1155/2013/972370

**Published:** 2013-06-03

**Authors:** Yue Guan, Ying Yin, Yan-Rong Zhu, Chao Guo, Guo Wei, Jia-Lin Duan, Yan-Hua Wang, Dan Zhou, Wei Quan, Yan Weng, Miao-Miao Xi, Ai-Dong Wen

**Affiliations:** Department of Pharmacy, Xijing Hospital, Fourth Military Medical University, Xi'an 710032, China

## Abstract

Traditional Chinese medicine uses a systemic treatment approach, targeting multiple etiological factors simultaneously. Danhong injection (DHI), a very popular Chinese medicine injection, is reported to be effective for many cardiovascular conditions. The primary active ingredients of DHI, and their systemic and interrelated mechanism have not been evaluated in an established myocardial ischemia/reperfusion (MI/R) model. We identified the main active constituents in DHI, including hydroxysafflor yellow A (A), salvianolic acid B (B), and danshensu (C), by HPLC fingerprint analysis and assessed their effect on MI/R rats and cardiomyocytes. These 3 compounds and DHI all decreased the levels of IL-1, TNF-**α**, and MDA, increased those of IL-10 and SOD activity *in vivo* and *in vitro*, and had antiapoptotic effects, as shown by flow cytometric analysis and TUNEL assay. Moreover, these compounds increased phosphorylation of Akt and ERK1/2 in cardiomyocytes. Interestingly, we found compound A exerted a more prominent anti-inflammatory effect than B and C, by decreasing NF-**κ**B levels; compound B had more powerful antioxidative capacity than A and C, by increasing Nrf2 expression; compound C had stronger antiapoptotic ability than A and B, by lowering caspase-3 activity. Our results elucidate the mechanisms by which DHI protects against MI/R induced injury.

## 1. Introduction

Ischemic cardiomyopathy is a public health concern with a rising incidence that results in high morbidity and mortality worldwide [1]. The therapeutic strategies aimed at restoring blood flow to the ischemic myocardium are frequently used in clinical practice; these include thrombolysis and primary angioplasty [2]. However, the process of reperfusion can paradoxically reduce the beneficial effects of restored blood flow, and even exacerbate necrotic cell death, as evidenced by an extension of the infarct size [3]. It has been reported that diverse pathological factors, such as inflammation, oxidative stress, and apoptosis, are all involved in the pathological mechanism underlying myocardial ischemia/reperfusion (MI/R) injury [4–8]. Thus, multitargets therapeutic strategies for MI/R injury are urgently required. 

Traditional Chinese medicine (TCM) offers many advantages in this respect. In TCM, combination therapy is the most commonly employed therapeutic approach, and the construction of a prescription containing multiple drugs, namely, a formula, each with distinct mechanisms, aims to amplify the therapeutic efficacy and lessen the adverse effects of each individual agent [9]. Generally, multiple active ingredients are aimed at multiple targets, exerting a systemic effect [10]. An excellent example of such a formula is Danhong injection (DHI), which was awarded the first Chinese medicine patent gold medal in the year of 2010. This formula is a standardized water-soluble complex containing extracts of the traditional Chinese remedies Radix *Salvia miltiorrhiza* (Danshen) and Flos *Carthamus tinctorius *L. (Honghua) [11]; clinically, the formula is applied extensively to tens of millions of patients with vascular dementia [12], angina pectoris [13, 14], acute coronary syndrome [15], thromboangiitis obliterans [16], and so forth. According to an authoritative survey report from Guosen Securities, China [17], DHI showed 3 consecutive annual sales of more than 1 billion yuan RMB, reaching 3 billion in 2011, and has become the top Chinese medicine for cardiovascular and cerebrovascular diseases in China. However, unclearness of effective components and functional mechanism of most TCM agents, especially formulae, limits their modernization and acceptance by Western medicine. Although many investigations have identified the active compounds and demonstrated the properties of salvia and safflower individually, the primary active ingredients in the mixed extraction formula that make up DHI, and their systemic and interrelated actions in an established cardiovascular disease model have not yet been clarified. The present study was designed to dissect the rationale for the formulation of DHI, to identify the main active ingredients, and, reveal to their mechanisms of action by using both experimental rat model of acute MI/R and simulated condition in primary cultured cardiomyocytes. 

## 2. Materials and Methods

### 2.1. HPLC Fingerprint

An Agilent 1100 series system (Agilent Corporation, USA) with a diode-array detector was used for chromatographic analysis, on an Agilent Zorbax Extend C18 reserved-phase analytical column (250 mm × 4.6 mm, 5 *μ*m, Agilent Corporation). The mobile phase was composed of solutions A (acetonitrile) and B (1% glacial acetic acid aqueous solution), and a gradient elution was performed (0–10 min, 8–10% A; 10–20 min, 10–17% A; 20–30 min, 17–22% A; 30–65 min, 22–30% A; 65–80 min, 22–30% A). There was a 15 minute re-equilibration between individual runs to recover the initial conditions. The column temperature was maintained at 25°C, and the detection wavelength was set at 280 nm. The flow rate was 1.0 mL/min and the injection volume was 20 *μ*L. 

DHI was obtained from Shaanxi Buchang Pharmaceutical Co., Ltd (China). Standard substances of hydroxysafflor yellow A (lot number: 111637-200905), salvianolic acid B (lot number: 111562-200908), Danshensu (lot number: 110855-201210), protocatechuic aldehyde (lot number: 110810-201007), and rosmarinic acid (lot number: 111871-201203) were purchased from the Chinese National Institute for the Control of Pharmaceutical and Biological Products.

### 2.2. Animals and Myocardial Ischemia/Reperfusion Injury

Sprague-Dawley (SD) rats (250–300 g) were purchased from the Laboratory Animal Institute of Fourth Military Medical University, Xi'an, China. Animals were anesthetized with sodium pentobarbital (60 mg/kg, intraperitoneally) and the chest opened by left thoracotomy. After the pericardium was incised, a 6–0 ligature was placed underneath the left anterior descending coronary artery (LAD). A small plastic snare was threaded through the ligature and placed in contact with the heart. Thereafter, the artery was occluded by applying tension to the ligature. After 30 min of ischemia, the ligature was released and rats were randomly administered by one of the following treatments intravenously: (1) saline; (2) major compounds of DHI (equal to the contents of DHI); (3) a combination of the major compounds of DHI (equal to the contents of DHI); (4) DHI. Sham-operated control rats underwent the same surgical procedures except for left anterior descending coronary artery occlusion. The injection volume was 2 mL/kg, throughout. After a 3 h reperfusion, blood samples were collected and diagnostic kits were used to detect serum levels of CK-MB, cTnI, IL-1, IL-10, TNF-*α* (Roche Molecular Biochemicals, Germany), SOD, and MDA (Nanjing Jiancheng Bioengineering Institute, China) according to the manufacturers' instructions.

### 2.3. Determination of Myocardial Infarct Size and Hemodynamic Parameters

 The infarct size was assessed with 3% Evans Blue (Sigma, USA) and 2% Triphenyltetrazolium Chloride (TTC, Sigma) staining as previously described [18]. Briefly, at the end of reperfusion, 2 mL of Evans blue dye (3% in saline) was injected into the jugular vein to delineate the ischemic zone from the nonischemic zone. The heart was rapidly excised and cross-sectioned into 6 slices. Each slice of the left ventricle (LV) was then counterstained with 2% TTC for 10 min at 37°C. After overnight storage in 4% paraformaldehyde, the slices were photographed. The area at risk (AAR) and infarct size (IS) were analyzed using Image-Pro Plus software (Media Cybernetics, USA). AAR was expressed as percentages of the left ventricular area (AAR/LV). IS was shown as percentages of the AAR (IS/AAR). The left ventricular end-diastolic pressure (LVEDP), first derivative of the left ventricular pressure (±dP/dt_max⁡_), and heart rate (HR) were obtained by the use of BL-420S Data Acquisition & Analysis System (Chengdu TME Technology, China).

### 2.4. Histopathological Examination

Myocardial tissue blocks were fixed in 4% paraformaldehyde and then embedded in paraffin. Serial sections were cut and stained with hematoxylin-eosin (H&E). The sections were analyzed under light microscope. The myonecrosis, inflammatory cell infiltration, and edema were evaluated in the section. 

### 2.5. Neonatal Cardiomyocytes Culture and Simulated Ischemia/Reperfusion (SI/R) Injury

Primary cultures of neonatal rat cardiomyocytes from 1- to 2-day-old SD rats were prepared and cultured as described previously [19]. The cells were suspended in Dulbecco's Modified Eagle's Medium (DMEM, Gibco, USA) containing 10% fetal calf serum (Gibco) and 0.1 mM 5′-bromo-2′-deoxyuridine and cultured at 37°C in a 5% CO_2_ incubator for 72 h. Cells were then pretreated with DHI (2%, v/v), or major compounds (equal to the contents in DHI), with or without 10 nM wortmannin (W, Sigma), an inhibitor of PI3K [20] or 10 *μ*M of U0126 (U, Sigma), an inhibitor of ERK [21] for another 24 h. Thereafter, SI/R was performed; cardiomyocytes were transferred into an ischemic buffer containing (in mmol/L) NaCl 137, KCl 3.8, MgCl_2_ 0.49, CaCl_2_ 0.9, HEPES 4, deoxyglucose 10, KCl 12, and lactate 20, pH 6.5, and were then incubated in a hypoxic/ischemic chamber (Billups-Rothenberg, USA) with 5% CO_2_ and 95% nitrogen, at 37°C for 2 h. After simulated ischemia, cells were returned to DMEM medium and were then incubated in a 5% CO_2_ incubator for 24 h. After reperfusion, the culture medium of the different treatment groups was collected for measurement of LDH (Nanjing Jiancheng Bioengineering Institute, China), IL-1, IL-10, TNF-*α*, and MDA levels; cell lysates were used to detect SOD and caspase-3 (Beyotime Institute of Biotechnology, China) expression.

### 2.6. Analysis of Cell Viability

Cell viability was measured using the 3-(4,5-dimethylthiazol-2-yl)-2, 5-diphenyl tetrazolium (MTT, Sigma, USA) assay. Cells were lysed in 150 *μ*l DMSO after incubation with MTT for 4 h and the amount of MTT formazan was quantified by determining the absorbance at 490 nm, using a microplate reader (Thermo Scientific, USA). Cell viability was expressed as a percentage of the control culture value.

### 2.7. Determination of Myocardial Apoptosis

To quantify the level of apoptosis, flow cytometry was performed. At the end of the hypoxic period, cells were washed and resuspended in binding buffer. Cells were then incubated for 10 min in the dark with 5 *μ*L Annexin V-FITC and 5 *μ*L propidium iodide (PI, KeyGEN, China) and analyzed with flow cytometry (BD Biosciences, USA). Apoptotic cells were also identified by the distinctive condensed or fragmented nuclear structure within cells stained with the terminal deoxynucleotidyl transferase-mediated dUTP nick end-labeling (TUNEL) assay (KeyGEN). The number of TUNEL-positive cells was presented as a percentage of the total cardiomyocytes.

### 2.8. Western Blotting Analysis

Proteins were extracted from myocytes and fractionated by 10% sodium dodecyl sulfate-polyacrylamide gel electrophoresis (SDS-PAGE) and then transferred onto PVDF membranes (Millipore, USA). The membranes were blocked with 5% nonfat dried milk and incubated overnight with primary antibodies (T-AKT, P-AKT, T-ERK, P-ERK, NF-*κ*B, Nrf2, *β*-actin; Cell Signaling, USA) at 4°C. All primary antibodies were used at a dilution of 1 : 1000. Subsequently, membranes were incubated with secondary antibodies at a 1 : 5000 dilution at 37°C for 30 min. The blots were visualized with ECL-Plus reagent (Santa Cruz, USA) and analyzed with Quantity One System image analysis software (Bio-Rad, USA).

### 2.9. Statistical Analysis

Data were expressed as mean ± SD. Statistical analysis was assessed by analysis of variance (ANOVA) followed by LSD test or Dunnett's test for multiple comparisons. Data analyses were performed using the SPSS v.17.0 software package. Differences were considered significant at *P* < 0.05. 

## 3. Results

### 3.1. Primary Active Ingredients Analysis of DH

The excellent postmarketing therapeutic effects of DHI on various cardiovascular and cerebrovascular diseases have raised questions which ingredients in this TCM formulation actually exerts effects, and what mechanisms are involved; however, to date these questions have remained unclear.

**Figure 1:**
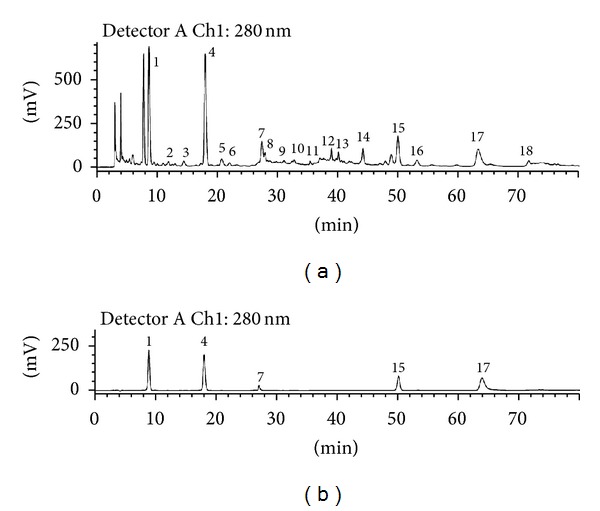
HPLC fingerprint of DHI. (a) 18 marked peaks are “common peaks” in DHI. (b) Standards for 1: Danshensu (C); 4: protocatechuic aldehyde (D); 7: hydroxysafflor yellow A (A); 15: rosmarinic acid (E); 17: salvianolic acid B (B).

Using established HPLC conditions, the HPLC profile of DHI was analyzed; the fingerprint of DHI is presented in Figure 1(a). Danshensu, protocatechuic aldehyde, hydroxysafflor yellow A, rosmarinic acid, and salvianolic acid B were identified by comparing the retention times and spectra with the relevant standards (Figure 1(b)). We analyzed the area under the curve to identify the major components; 4 ingredients from *Salvia miltiorrhiza* and 1 from *Carthamus tinctorius* were eventually identified. The components A, B, C, D, and E were identified as hydroxysafflor yellow A, salvianolic acid B, Danshensu, protocatechuic aldehyde, and rosmarinic acid, respectively. Their respective levels in DHI were determined as 24.0 ± 0.1, 728.8 ± 6.7, 1000.3 ± 9.3, 88.2 ± 0.4, and 205.2 ± 1.2 *µ*g/mL.

**Figure 2:**
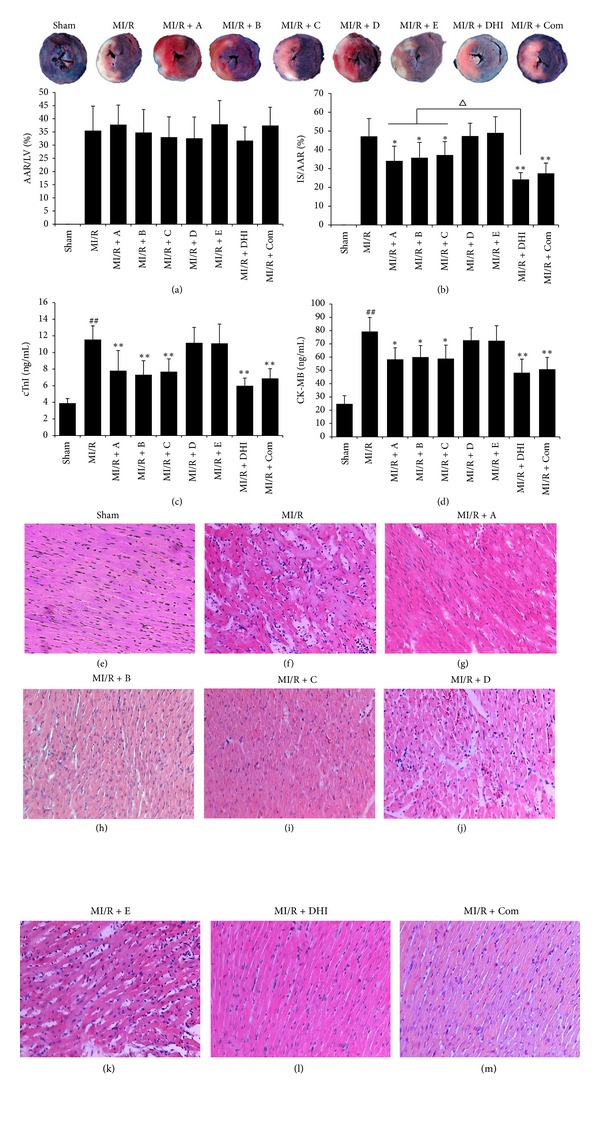
Determination of the major active ingredients in DHI involved in myocardial protection. (a) and (b) The percentage of AAR/LV and IS/AAR of different treatments. (c) and (d) Serum levels of biochemical indicators cTnI and CK-MB of each group. (e) and (m) Representative light microscopic (H&E, ×40) appearances of rat myocardial histopathological morphology for each group. AAR, area at risk; LV, left ventricle; IS, infarct size; MI/R, myocardial ischemia/reperfusion; A, hydroxysafflor yellow A; B, salvianolic acid B; C, danshensu; D, protocatechuic aldehyde; E, rosmarinic acid; DHI, Danhong injection; Com, the combination of A, B, C, D, and E. Data are presented as mean ± SD (*n* = 6). Solid circles represent individual results. ^##^ *P* < 0.01 versus control; *P < 0.05, **P < 0.01 versus MI/R; ^△^ *P* < 0.05 versus MI/R + DHI.

To test whether compounds A, B, C, D, and E had an effect equal to that of DHI, the combination of these 5 individual components was administered to MI/R rats. Under the premise that AAR/LV was similar among groups (Figure 2(a)), a 3 h reperfusion resulted in IS/AAR enhancement in MI/R group. The myocardial IS of the AAR in the group receiving the combination or DHI was not significantly different (Figure 2(b)), which indicated that the combined effect of A, B, C, D, and E could account for the effect of DHI. Administration of A, B, C, DHI, or the combination significantly reduced IS compared to that of in MI/R group, while D and E showed no contribution. However, among the effective treatments, DHI and the combination were more effective than A, B, or C. 

Hemodynamic data for HR, LVEDP, +dP/dt_max⁡_, and −dP/dt_max⁡_ at the end of reperfusion were also determined to confirm the result obtained by analysis of IS. There were no significant differences in HR among these groups. However, MI/R process significantly impaired LVEDP and ±dP/dt_max⁡_. Treatment with A, B, C, DHI, or the combination, significantly decreased the LVEDP and increased ±dP/dt_max⁡_, while no changes were observed in groups treatment with D or E compared to MI/R group (data not shown).

We measured serum levels of CK-MB and cTnI in order to confirm the previous results. At the end of reperfusion, both indicators were significantly lowered in treatment groups compared to those of treatment with saline. But D and E displayed no protection on MI/R injury, while treatment with the combination showed a similar effectiveness to that of DHI (Figures 2(c) and 2(d)).

Heart tissue samples from the group treatment with saline, D, or E showed more widespread disorder of myocardial structure, diffuse cloudy swelling, as well as infiltration of inflammatory cells (Figures 2(e)–2(m)). Treatment with A, B, C, DHI, or the combination significantly ameliorated the myocardial damage caused by 30 min ischemia and 3 h reperfusion.

Taken together, the above results indicate that the combined effect of A, B, C, D, and E could represent the effect of DHI, and the individual components A, B, and C may be the main effective components of DHI. 

### 3.2. Pretreated A, B, C, and DHI Attenuated the SI/R-Induced Cardiomyocytes Injury

**Figure 3:**
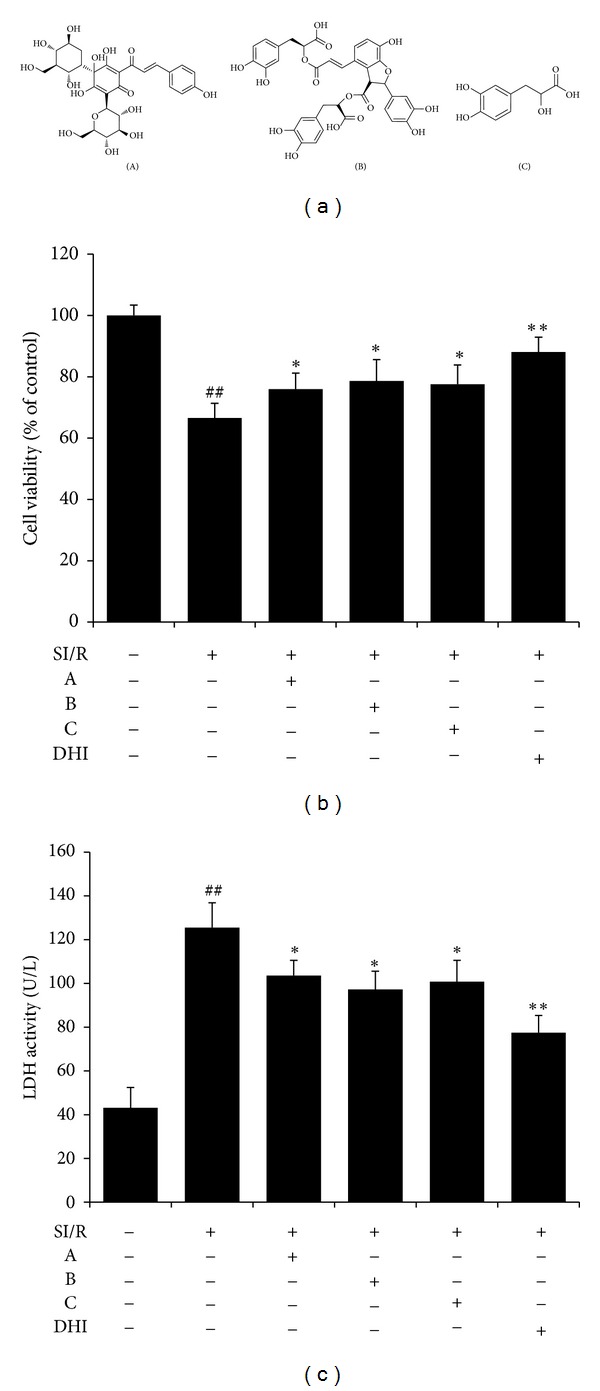
Protective effect of A, B, C, and DHI on SI/R-induced cytotoxicity in neonatal rat cardiomyocytes. (a) Chemical structures of A, B, and C, respectively. (b) Effects of A, B, C, and DHI on SI/R-induced reduction of cell viability. (c) Effects of A, B, C, and DHI on LDH activity in the cultured supernatants of cardiomyocytes subjected to SI/R. LDH, lactate dehydrogenase; SI/R, simulated ischemia/reperfusion; A, hydroxysafflor yellow A; B, salvianolic acid B; C, danshensu; DHI, Danhong injection. Data are presented as mean ± SD of 3 independent experiments. ^##^ *P* < 0.01 versus control; *P < 0.05,**P < 0.01 versus SI/R.

 The chemical structures of A, B, and C are shown in Figure 3(a). To investigate the protective effects of these 3 effective constituents as well as that of DHI on cardiomyocytes, an MTT assay was performed. SI/R induced a significant decrease in cell survival compared to control cells. Treatment with A, B, C, and DHI prevented cells from SI/R damage, restoring cell survival (Figure 3(b)). As shown in Figure 3(c), LDH activity in the cultured supernatant significantly increased in the SI/R group. Pretreatment with A, B, C, and DHI markedly reduced the activity of LDH of cardiomyocytes subjected to SI/R. 

### 3.3. The Anti-Inflammatory Effect of A, B, C, and DHI

IL-1, IL-10, and TNF-*α* are well-known inflammatory cytokine markers, and the effect of the various treatments on the levels of these cytokines was analyzed *in vivo* and *in vitro*. As shown in Table 1, the levels of IL-1 and TNF-*α* were significantly elevated in both the MI/R and SI/R group and were markedly decreased by treatment with A, B, C, or DHI. Similarly, IL-10 production rose notably after treatment with DHI or its major active ingredients. In addition, our data indicated that DHI and A had more potent anti-inflammatory effects than B and C. Importantly, the anti-inflammatory characteristics of A, B, C, and DHI were significantly blocked by the PI3K inhibitor wortmannin and ERK inhibitor U0126 in SI/R induced cardiomyocytes.

### 3.4. The Antioxidative Effect of A, B, C, and DHI

To evaluate the level of oxidative stress, MDA and SOD activities were detected in serum as well as in the cultural supernatant. As shown in Table 1, after reperfusion, MDA production was markedly increased compared with MDA production in the nonischemia/reperfused group, while SOD was decreased. Treatment with A, B, C, or DHI substantially reversed the increase in MDA production, as well as the decrease of SOD activity. Our results also showed that the antioxidant effects displayed by A and C were clearly inferior to those of B and DHI. Moreover, wortmannin and U0126 treatment significantly inhibited the antioxidant activity of DHI and its main active constituents, both in rat serum and in the supernatants of cultured cardiomyocytes after I/R injury.

### 3.5. The Antiapoptotic Effect of A, B, C, and DHI

**Figure 4:**
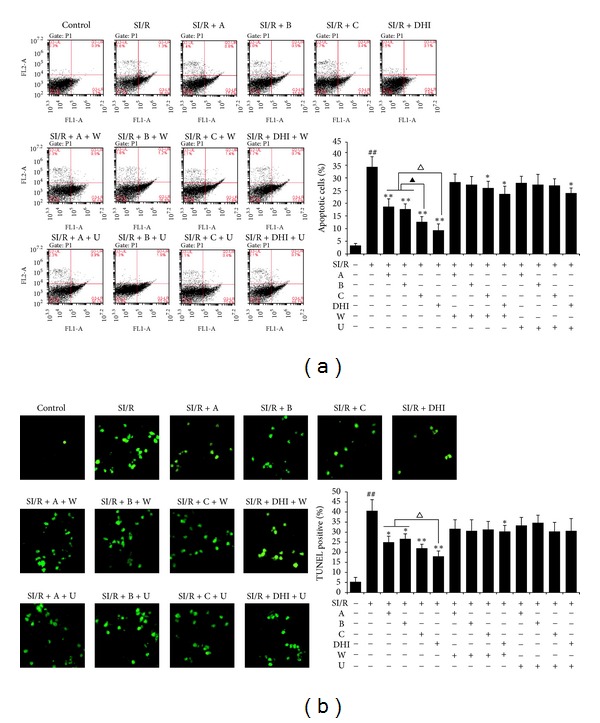
Flow cytometric analysis (a) and TUNEL staining (b) to detect the apoptotic ratio of cardiomyocytes exposed to different treatments followed by SI/R. Images: TUNEL-positive myocytes (stained green, ×40 objective). SI/R, simulated ischemia/reperfusion; A, hydroxysafflor yellow A; B, salvianolic acid B; C, danshensu; DHI, Danhong injection; W, wortmannin; U, U0126. Data are presented as mean ± SD of 3 independent experiments. ^##^ *P* < 0.01 versus control group; *P < 0.05, **P < 0.01 versus SI/R group; ^▲^ *P* < 0.01 versus SI/R + C group; ^△^ *P* < 0.05 versus SI/R + DHI group.

As shown in Figure 4, after 24 h of reperfusion, the apoptotic index was significantly increased in the SI/R group compared to that in the control group, whereas the apoptotic index was significantly decreased by treatment with A, B, C, or DHI, compared with that of the SI/R group. Interestingly, the antiapoptotic effect of DHI was markedly stronger than that of its constituents, and flow cytometric analysis showed that C played a stronger role in inhibiting SI/R-induced apoptosis in cardiomyocytes than did A and B. Furthermore, simultaneous treatment with either wortmannin or U0126 significantly attenuated the antiapoptotic effect afforded by A, B, C, and DHI, in cardiomyocytes subjected to SI/R. 

### 3.6. A, B, C, and DHI Enhanced Phosphor-Akt and Phosphor-ERK1/2 in Primary Cultured Cardiomyocytes

**Figure 5:**
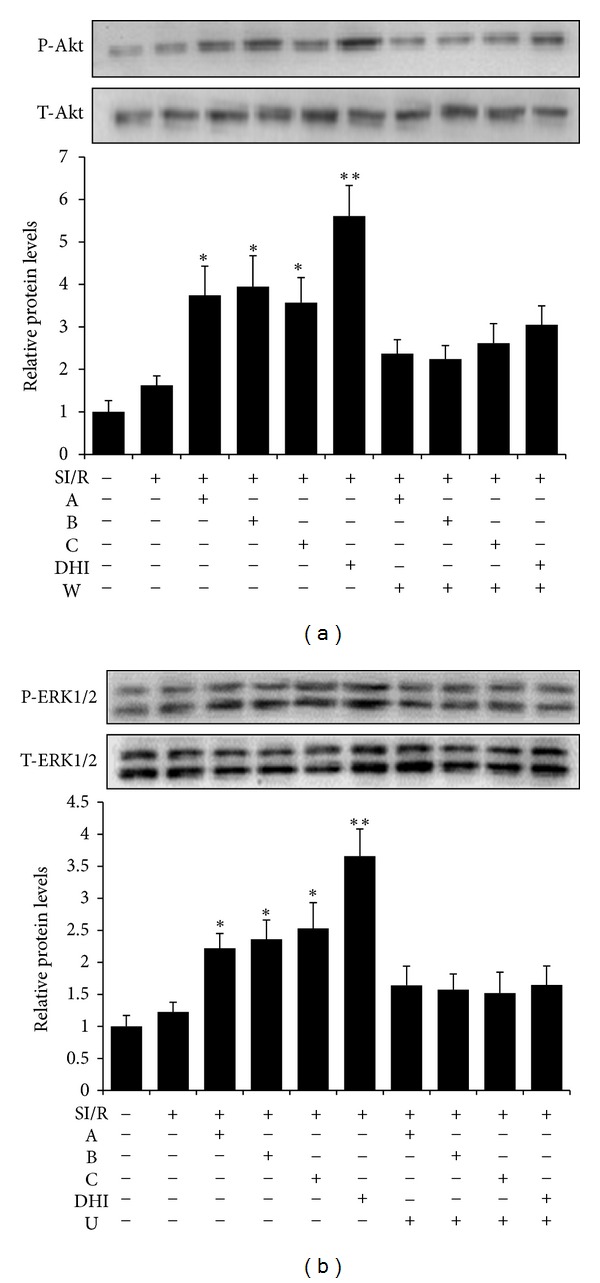
DHI and its major active ingredients upregulated expression of phospho-Akt (a) and phospho-ERK1/2 (b) in cardiomyocytes exposed to SI/R. SI/R, simulated ischemia/reperfusion; A, hydroxysafflor yellow A; B, salvianolic acid B; C, danshensu; DHI, Danhong injection; W, wortmannin; U, U0126. Data obtained from quantitative densitometry are presented as mean ± SD of 3 independent experiments. *P < 0.05, **P < 0.01 versus SI/R.

Well-known Reperfusion Injury Salvage Kinase (RISK) signaling pathways, including Akt and ERK1/2 signaling pathways, are involved in cardioprotection [2]. Studies over the past decades have confirmed the importance of Akt and ERK1/2 in the regulation of cell survival and proliferation. In the present study, western blot analysis was used to detect phosphor-Akt and phosphor-ERK1/2 in primary cardiomyocytes. As shown in Figure 5, pretreatment with A, B, C, or DHI markedly upregulated-phosphorylation of Akt and ERK1/2, which was abolished by simultaneous treatment with wortmannin or U0126. 

### 3.7. Pretreatment of A, B, C, and DHI Modulated the Levels of NF-*κ*B, Nrf2, and Caspase-3

**Figure 6:**
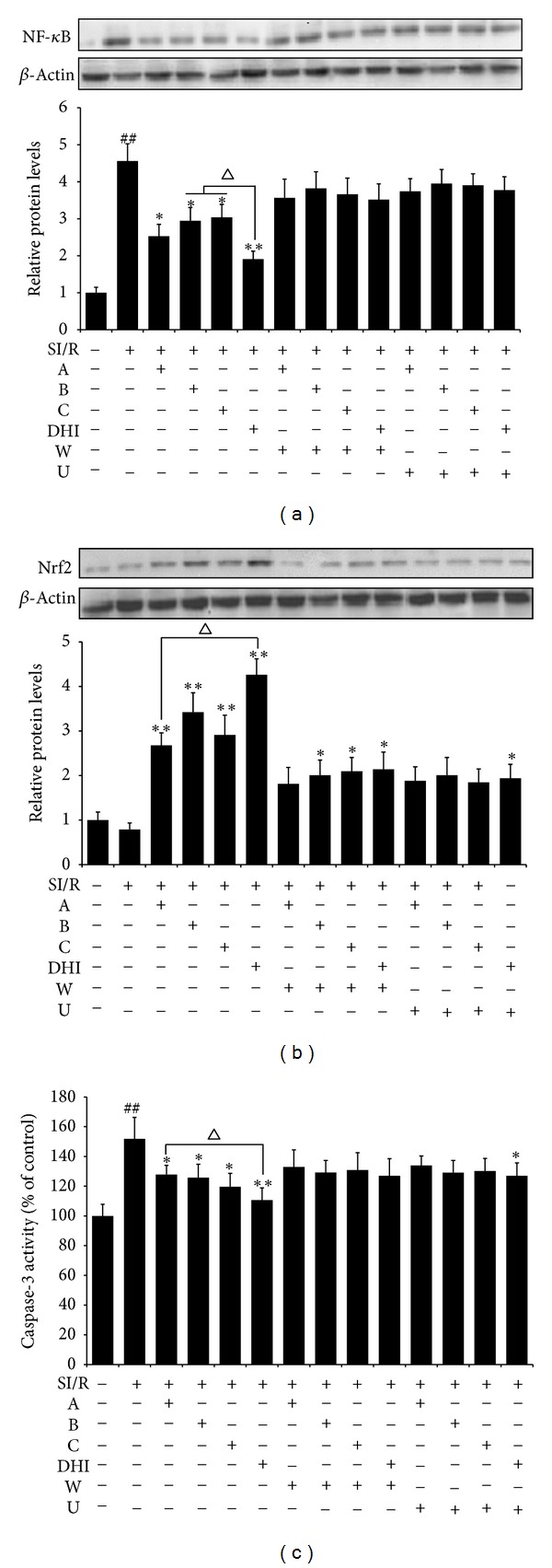
Effects of pretreatment with A, B, C, and DHI on the levels of NF-*κ*B (a), Nrf2 (b), and Caspase-3 (c) in neonatal rat cardiomyocytes subjected to SI/R. SI/R, simulated ischemia/reperfusion; A, hydroxysafflor yellow A; B, salvianolic acid B; C, Danshensu; DHI, Danhong injection; W, wortmannin; U, U0126. Data are presented as mean ± SD of at least 3 independent experiments. ^##^ *P* < 0.01 versus control; *P < 0.05, **P < 0.01 versus SI/R.

To explore the mechanisms responsible for the beneficial effects of A, B, C, and DHI, we measured the expression levels of NF-*κ*B, Nrf2, and Caspase-3, which play a crucial role in inflammation [22], oxidative damage [23], and apoptosis [24], respectively. As shown in Figures 6(a) and 6(c), administration of A, B, C, or DHI, individually, significantly downregulated the expression levels of NF-*κ*B and Caspase-3 that had been upregulated by SI/R. Moreover, to some extent, cells treated with A showed more effective inhibition of NF-*κ*B expression, whereas cells treated with C showed greater reduction of Caspase-3 activity compared with cells treated with the other 2 individual components. However, the downregulation of NF-*κ*B and Caspase-3 by these active components was almost completely blocked by pretreating cells with wortmannin or U0126. In contrast, cells treatment with A, B, or C significantly increased the expression of Nrf2 compared with the SI/R group (Figure 6(b)); the most marked effect, however, was observed in the DHI-treated cells. Moreover, compared with A and C, B had a more marked effect on increasing Nrf2 expression. As before, pretreatment with wortmannin or U0126 abrogated the upregulation of Nrf2 expression to a great extent. These observations indicated that, on the one hand, all 3 of the active components had a pronounced anti-inflammatory, antioxidative, and antiapoptotic effects, but, on the other hand, each compound also had particular advantages in terms of these protective effects. Moreover, these cardioprotective mechanisms may involve the activation of PI3K/ Akt and ERK pathways. 

## 4. Discussion

The excellent postmarketing therapeutic effects of DHI on various cardiovascular and cerebrovascular diseases have raised questions which ingredients in this TCM formulation actually exerts effects, and what mechanisms are involved; however, to date these questions have remained unclear. The present study for the first time has examined the active components in DHI using HPLC-fingerprinting in combination with animal experimentation and explored the multiple molecular mechanisms of the main components in terms of presumed underlying effects, such as inflammation, oxidative stress, and apoptosis. We have demonstrated that compounds A, B, and C of DHI are the main active ingredients; all 3 play protective roles against MI/R injury, via synergistic anti-inflammatory, antioxidative, and antiapoptotic effects involving the RISK pathway. Our results elucidate the mechanisms that are responsible for DHI protecting against MI/R induced injury.

Investigating the spectral fingerprint could improve component analysis of TCM. The DHI fingerprint obtained in this study differed from the published DHI chromatogram [11, 25], most probably owing to the use of distinct chromatographic conditions. By analyzing the concentration ratios against standard substances, those components with the highest concentration in *Salvia miltiorrhiza* and *Carthamus tinctorius* were determined as the potential active ingredients, which were then used for pharmacodynamic screening. Although protocatechuic aldehyde and rosmarinic acid are reported to have cardioprotective effects against I/R injury [26, 27], this was discrepant to our results; the reason may be related to the concentration of these substances, since the administrated dosage in the present study was adjusted according to their respective levels in DHI. Finally, compounds A, B, and C were prioritized for further research following the beneficial results. Their protective functions were judged by the gold standard for MI/R cardioprotection, such as infarct size [28], CK-MB [29], and cTnI [30] levels.

It is now widely recognized that the process of I/R injury is characterized by inflammatory lesions, oxidative damage, and apoptosis, more importantly cross-talk among these factors. Inflammation is closely associated with I/R injury, which is mediated by excessive generation of reactive oxygen species (ROS) or oxidative stress [31]. In order to determine whether the anti-inflammatory activity of DHI and its main active ingredients are associated with the protective role against I/R injury, we have measured the levels of cytokines like proinflammatory cytokines TNF-*α*, IL-1 [32], and myocardial protective cytokines IL-10 [33]. MI/R resulted in a significant increase in IL-1 and TNF-*α* levels, and a significant decrease in that of IL-10. These MI/R-induced changes in inflammation-related indexes were inhibited by the administration of A, B, C, and DHI. Interestingly, compared with the effects of B and C, A showed more effective in anti-inflammatory properties, indicating that A is the main anti-inflammatory composition in DHI. Furthermore, it is known that NF-*κ*B, a transcription factor, once activated, translocates into the nucleus and binds to DNA to induce the expressions of various genes, including IL-1, IL-10, and TNF-*α*, which are critical for cell survival, inflammation, and immunity [22, 34]. The current study showed that upregulation of NF-*κ*B expression in cardiomyocytes after I/R could be inhibited by A, B, C, and DHI in parallel with a decrease in IL-1 and TNF-*α* levels and an increase in IL-10. These results provide evidence that the anti-inflammatory activity of DHI in I/R-induced myocardial injury could be due to the regulation of the inflammation mediators via NF-*κ*B pathway. 

On the other hand, oxidative stress mediators, such as ROS released by inflammatory cells around I/R injured areas, are also suggested to play a critical role in MI/R injury [35]; high concentrations of ROS can inhibit cell proliferation and induce apoptosis [21]. A number of antioxidant enzymes are responsible for the removal of excess ROS in living organism. Among these, SOD is the most crucial enzymes in the cellular antioxidant system [36]. Furthermore, as an important product of lipid peroxidation, MDA also indirectly reflects the production of intracellular ROS [23]. In the present study, the levels of SOD and MDA were measured both in serum of rats and in cultural supernatant of cardiomyocytes. Our results demonstrated that A, B, C, and DHI, more specially B, could significantly inhibit MI/R-induced oxidative stress, thus contributing to the attenuation of MI/R injury. Nrf2 is a transcription factor that regulates an expansive set of antioxidant-related genes, which act in synergy to remove ROS through sequential enzymatic reactions [23]. Among the spectrum of anti-oxidant genes, expression of those encoding catalase, SOD, glutathione reductase, and glutathione peroxidase is all controlled by Nrf2 [37]. To further support our findings, the expression of Nrf2 was determined by western blot assay. In cardiomyocytes that underwent SI/R, we found that Nrf2 activity was significantly enhanced by treatment with DHI and its major active constituents. Therefore, we can conclude that A, B, C, and DHI inhibited the oxidative damage induced in cardiomyocytes by I/R through upregulation of Nrf2 expression and subsequent suppressing oxidative stress. 

Apoptosis plays a critical pathogenic role in MI/R injury. Previous studies reported that oxygen-free radicals and inflammatory cytokines could regulate cell apoptosis [38, 39]. Here, apoptosis in cardiomyocytes was analyzed using flow cytometric and TUNEL staining. SI/R significantly increased myocytes apoptosis, whereas treatment with A, B, C, and DHI markedly reduced the apoptosis, indicating that DHI and its main active ingredients protected against SI/R-induced apoptosis. We also found that of the 3 compounds, C plays the strongest antiapoptotic role in the effect of DHI. Caspase-3 is one of the most pivotal effector caspases that is sequentially activated, autocatalyzed, and processed into activated fragments during apoptosis [24]. Our study confirmed that induction of apoptosis by SI/R was associated with activation of caspase-3, while incubation with A, B, C, or DHI resulted in attenuated caspase-3 activation.

The RISK pathway contains a group of survival protein kinases, including Akt and ERK1/2, which confer powerful cardioprotection against lethal MI/R injury. After the concept of the RISK pathway was originally conceived, it was supported by many studies reporting the infarct-limiting effect of pharmacological activation of the RISK pathway, which was associated with mediating a form of programmed cell survival, thereby preventing I/R injury [40, 41]. In addition, both Akt and ERK1/2 signaling are involved in the cardiovascular protective mechanisms of anti-inflammation and antioxidation. In the present study, we used inhibitors of PI3K-Akt (wortmannin) and ERK1/2 (U0126) pathways to test the hypothesis that the action of DHI and its active constituents is mediated through one or more components of the RISK pathway. Our results demonstrated that both wortmannin and U0126 could block the cardioprotective effect of DHI and its active constituents against MI/R, which indicated that DHI, as well as A, B, and C could activate the RISK pathway to exert myocardial protection.

**Figure 7:**
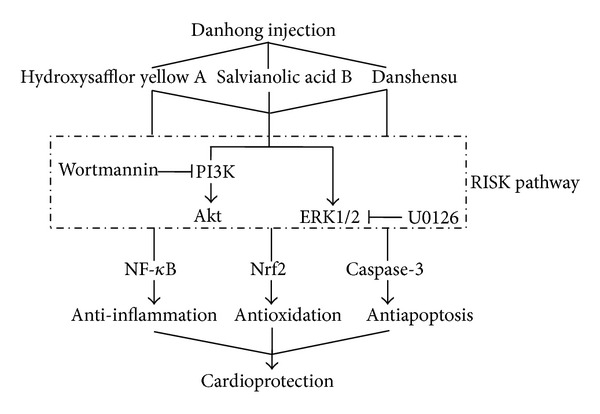
Proposed cardioprotective effects and potential mechanisms of the active ingredients of Danhong injection.

In summary, our study demonstrated that hydroxysafflor yellow A, salvianolic acid B, and danshensu are the main active compounds of DHI, and they ameliorate MI/R injury via anti-inflammatory, antioxidative, and antiapoptotic effects by individually activating the RISK pathway (Figure 7). Our findings indicate that the DHI formula possibly exerts its therapeutic polypharmacology through systematic drug combinations hitting multiple targets, thereby contributing to the overall effectiveness of the treatment. This research combined molecular, cellular, and animal approaches to reveal the mechanism of action of DHI in MI/R injury therapy. Although those constituents presenting at low levels in DHI were not involved in this research, we proposed an effective strategy to find new combinations for the systematic treatment of complex diseases, directed at multiple targets based on TCM principles. In the future, the effects of the low-content constituents in DHI on MI/R injury should be investigated.

## Figures and Tables

**Table 1:** Levels of IL-1, IL-10, TNF-*α*, MDA, and SOD in serum of rats and in culture supernatants of cardiomyocytes exposed to different treatments (mean ± SD, *n* = 6).

Treatment	Inflammatory cytokines markers			Oxidative stress markers	
	IL-1 (pg/mL)	IL-10 (pg/mL)	TNF-*α* (pg/mL)	MDA (nmol/mL)	SOD (U/mL)
Serum					
Sham	130.3 ± 43.4	146.2 ± 5.9	103.8 ± 8.4	11.8 ± 1.8	290.9 ± 15.4
MI/R	391.1 ± 57.6^##^	159.0 ± 11.4	279.6 ± 13.7^##^	23.5 ± 3.2^##^	196.9 ± 12.0^##^
MI/R + A	255.2 ± 37.5**	298.8 ± 28.6**	131.1 ± 21.3**	16.7 ± 2.6^∗§△^	224.7 ± 15.5^∗§△^
MI/R + B	326.9 ± 48.2^∗†△^	208.7 ± 20.1^∗†△^	185 ± 33.2^∗†△^	8.2 ± 2.1**	252.3 ± 11.9**
MI/R + C	362.7 ± 53.4^∗†△^	211.0 ± 20.2^∗†△^	171 ± 25.0^∗†△^	18.5 ± 3.2^∗§△^	223.2 ± 16.9^∗§△^
MI/R + DHI	252.4 ± 49.7**	315.0 ± 42.2**	128 ± 31.4**	6.9 ± 3.2**	258.2 ± 17.1**
Culture supernatant					
Control	65.91 ± 7.3	56.0 ± 11.1	22.1 ± 5.9	1.1 ± 0.1	26.6 ± 2.2
SI/R	165.7 ± 11.5^##^	115.7 ± 12.4^##^	93.9 ± 14.1^##^	2.9 ± 0.3^##^	15.5 ± 2.5^##^
SI/R + A	102.0 ± 8.6**	172.0 ± 15.6**	44.0 ± 12.8*	2.1 ± 0.2^∗§△^	21.0 ± 1.9^∗△^
SI/R + B	129.5 ± 14.0^∗†△^	156.4 ± 14.1^∗△^	54.7 ± 13.7^∗△^	1.6 ± 0.2**	23.3 ± 1.1**
SI/R + C	132.3 ± 11.9^∗†△^	150.9 ± 13.6^∗△^	52.7 ± 12.2^∗△^	2.0 ± 0.3^∗△^	20.1 ± 1.3^∗§△△^
SI/R + DHI	87.1 ± 14.1**	194.2 ± 17.4**	29.4 ± 6.8**	1.3 ± 0.2**	24.8 ± 1.1**
SI/R + A + W	156.5 ± 12.8	135.2 ± 18.3	71.9 ± 10.8	2.6 ± 0.4	17.1 ± 2.5
SI/R + B + W	157.6 ± 11.9	129.9 ± 21.0	69.5 ± 13.6	2.6 ± 0.5	18.1 ± 2.2
SI/R + C + W	144.7 ± 13.3	124.4 ± 13.5	80.5 ± 15.1	2.5 ± 0.2	17.9 ± 1.3
SI/R + DHI + W	149.5 ± 15.8	140.0 ± 11.6	66.1 ± 11.3	2.4 ± 0.2	19.1 ± 1.1
SI/R + A + U	151.4 ± 15.1	137.3 ± 19.1	73.3 ± 8.7	2.5 ± 0.5	17.4 ± 2.2
SI/R + B + U	155.6 ± 17.4	130.7 ± 14.6	63.9 ± 13.8	2.7 ± 0.4	19.0 ± 2.4
SI/R + C + U	152.4 ± 10.2	127.9 ± 13.6	72.3 ± 12.6	2.6 ± 0.2	18.2 ± 1.3
SI/R + DHI + U	146.6 ± 12.5	133.0 ± 16.6	64.6 ± 14.0	2.3 ± 0.2	19.3 ± 1.6

MI/R: myocardial ischemia/reperfusion; SI/R: simulated ischemia/reperfusion; A: hydroxysafflor yellow A; B: salvianolic acid B; C: danshensu; DHI: Danhong injection; W: wortmannin; U: U0126; IL-1: interleukin 1; IL-10: interleukin 10; TNF-*α*: tumor necrosis factor-alpha; MDA: malondialdehyde; SOD: superoxide dismutase. ^##^ *P* < 0.01 versus Sham or Control; *P < 0.05, **P < 0.01 versus MI/R or SI/R; ^†^ *P* < 0.05 versus MI/R + A or SI/R + A; ^§^ *P* < 0.05 versus MI/R + B or SI/R + B; ^△^ *P* < 0.05, ^△△^ *P* < 0.01 versus MI/R + DHI or SI/R + DHI.
